# Targeted cytotoxic analog of luteinizing hormone-releasing hormone (LHRH), AEZS-108 (AN-152), inhibits the growth of DU-145 human castration-resistant prostate cancer *in vivo* and *in vitro* through elevating p21 and ROS levels

**DOI:** 10.18632/oncotarget.2146

**Published:** 2014-06-29

**Authors:** Petra Popovics, Andrew V. Schally, Luca Szalontay, Norman L. Block, Ferenc G. Rick

**Affiliations:** ^1^ Veterans Affairs Medical Center and South Florida Veterans Affairs Foundation for Research and Education, Miami, FL; ^2^ Department of Pathology, University of Miami, Miller School of Medicine, Miami, FL; ^3^ Division of Hematology/Oncology, Department of Medicine, University of Miami, Miller School of Medicine, Miami, FL; ^4^ Division of Endocrinology, Department of Medicine, University of Miami, Miller School of Medicine, Miami, FL; ^5^ Division of Cardiovascular Diseases, Department of Medicine, University of Miami, Miller School of Medicine, Miami, FL; ^6^ Department of Urology, Herbert Wertheim College of Medicine, Florida International University, Miami, FL; ^7^ Department of Medicine III, Medical Faculty Carl Gustav Carus, Dresden, Germany

**Keywords:** cytotoxic peptide analog, targeted therapy, GnRH, reactive oxygen species, hormone-naive prostate cancer, CRPC, LHRH agonist

## Abstract

Management of castration-resistant prostate cancer (CRPC) is challenging due to lack of efficacious therapy. Luteinizing hormone-releasing hormone (LHRH) analogs appear to act directly on cells based on the LHRH receptors on human prostate adenocarcinoma cells. We explored anticancer activity of a cytotoxic analog of LHRH, AEZS-108, consisting of LHRH agonist linked to doxorubicin. Nude mice bearing DU-145 tumors were used to compare antitumor effects of AEZS-108 with its individual constituents or their unconjugated combination. The tumor growth inhibition of conjugate was greatest among treatment groups (90.5% inhibition vs. 41% by [D-Lys(6)]LHRH+DOX). The presence of LHRH receptors on DU-145 cells was confirmed by immunocytochemistry. In vitro, AEZS-108 significantly inhibited cell proliferation (61.2% inhibition) and elevated apoptosis rates (by 46%). By the detection of the inherent doxorubicin fluorescence, unconjugated doxorubicin was seen in the nucleus; the conjugate was perinuclear and at cell membrane. Autophagy, visualized by GFP-tagged p62 reporter, was increased by AEZS-108 (7.9-fold vs. 5.3-fold by DOX+[D-Lys(6)]LHRH. AEZS-108 more effectively increased reactive oxygen species (ROS, 2-fold vs. 1.4-fold by DOX+[D-Lys(6)]LHRH) and levels of the apoptotic regulator p21 in vivo and in vitro. We demonstrate robust inhibitory effects of the targeted cytotoxic LHRH analog, AEZS-108, on LHRHR positive castration-resistant prostate cancer cells.

## INTRODUCTION

In 2014, prostate cancer is expected to account for 27% of newly detected cancers and 10% of cancer-related deaths in men in the US [1]. Standard therapy of metastatic prostate cancer is androgen deprivation therapy (ADT) currently achieved by surgical orchiectomy or by treatment with LHRH agonist or antagonist [2-4]. Continuous administration of LHRH analogs downregulates pituitary LHRH receptors thus leading to the suppression of gonadotropin (luteinizing hormone and follicle-stimulating hormone) release and a consequent drop in production of gonadal androgens [2, 5]. However, the attenuation of this pituitary-gonadal axis is preceded by an initial surge of LH that, in some cases, is responsible for a serious side effect termed “flare reaction” [6, 7]. To avoid side effects, antagonistic analogs of LHRH, that completely lack LH-releasing activity, have also been developed and approved for the management of advanced androgen-dependent prostate cancer [8-10].

Despite the initial success of ADT on suppressing tumor growth, prostate cancer cells may progress to androgen independence (also referred to as castration resistance) which situation narrows the number of effective treatments. Patients with castrate resistant prostate cancer (CRPC) have a poor prognosis with an expected survival of less than 19 months [11]. There are a few treatment options of chemotherapy for advanced CRPC but none of them are markedly effective [12].

LHRH and its receptor (LHRH-R) are not limited to the hypothalamic-pituitary axis [13]. In the periphery, the LHRH system regulates gonadal functions and appears to serve as a growth factor of benign conditions [14-17] and various cancers including breast, lung, ovary, endometrial, kidney, bladder, colon, pancreas and prostate [18-25]. A specific, medium to high-affinity binding site for an LHRH agonist was found in 86% of prostate cancers [23]. In addition, expression of LHRH receptor by prostate cancer cells is preserved even after a prolonged exposure to LHRH agonist; LHRH receptors also appear in lymph node metastases [26]. These findings imply that the LHRH receptor is a suitable object for the design of an approach based on targeted chemotherapy [27]. Accordingly, various cytotoxic conjugates of LHRH have been produced of which AEZS-108 (previously known as AN-152) has been chosen for clinical development [28-30]. This compound consists of an agonistic analog of LHRH, [D-Lys^6^]-LHRH, linked to the cytotoxic anthracycline, doxorubicin. In this hybrid, doxorubicin remains active and becomes specifically targeted to cells that possess cell membrane LHRH receptors, thereby making it less toxic to other cells [31, 32]. The unconjugated doxorubicin diffuses through the cell membranes and accumulates in the nucleus where it intercalates into the DNA. By targeting topoisomerase-II, it triggers enzyme-mediated DNA damage [33, 34]. Furthermore, doxorubicin elevates the concentration of reactive oxygen species (ROS) and triggers the activation of the ceramide signaling pathway – both processes being antiproliferative and toxic to the cells [35-40]. In contrast, AEZS-108 is taken up by the cells by receptor internalization and the doxorubicin moiety is subsequently cleaved off in the cell [41, 42]. This suggests a delayed doxorubicin-induced cytotoxicity in cells that are otherwise sensitive to doxorubicin. However, previous findings showed that AEZS-108 is more effective in inducing apoptosis in ovarian and endometrial carcinoma cell lines *in vitro* than doxorubicin alone [43].

In the current study, we investigated the antitumor activity of AEZS-108 in the CRPC DU-145 tumors *in vivo* and *in vitro*. Additionally, we explored the mechanism of its action *in vitro*, by studying its intracellular dynamics, measuring its effect on apoptosis and autophagy, and detecting its potential to activate doxorubicin-mediated intra- and extranuclear signals. Our findings indicate that AEZS-108 is a highly promising compound for the treatment of CRPC not only due to its targeted nature but also because of the potential of the different uptake mechanism to strengthen the extranuclear toxic activity of doxorubicin.

## RESULTS

### Inhibition of tumor growth in nude mice by AEZS-108

**Figure 1:**
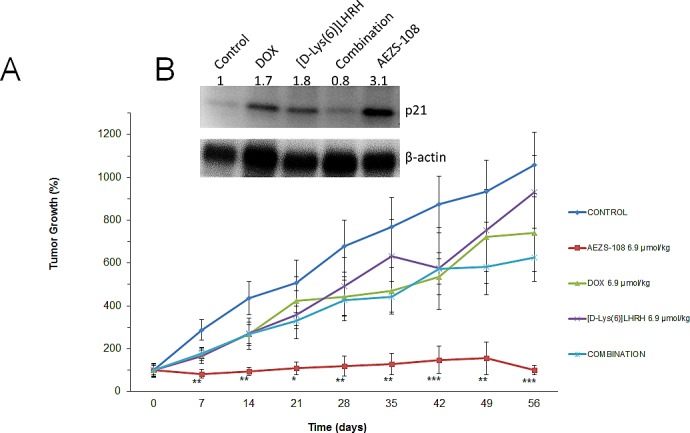
Treatment with cytotoxic luteinizing hormone-releasing hormone (LHRH) analog, AEZS-108, significantly inhibited the growth of DU-145 castration-resistant prostate cancer xenografted into nude mice and upregulated the protein levels of p21 WAF1/Cip1 A: Mice were given vehicle (control), 6.9 μmol/kg AEZS-108, 6.9 μmol/kg doxorubicin (DOX), 6.9 μmol/kg [D-Lys(6)]LHRH) or the combination of unconjugated DOX and [D-Lys(6)]LHRH in weekly i.v. injections. Tumor volumes were assessed each week and percent changes in tumor volumes are shown (± SEM) relative to tumor volumes at the initiation of treatment. Statistical analysis was performed by one-way ANOVA, followed by Holm-Sidak test.*: p < 0.05, **: p < 0.01, ***: p < 0.001 vs. control. B: Western blot showing protein levels of p21 WAF1/Cip1 in tumors collected at the end of the experiment. β-actin was used as loading control. Fold-changes in protein levels are shown relative to control and normalized to β-actin levels. Membrane shown is representative of two experiments.

Nude mice bearing xenografted tumors of castration-resistant prostate DU-145 cells were randomized into 5 treatment groups: control (vehicle), AEZS-108 (6.9 μmol/kg), DOX (6.9 μmol/kg), [D-Lys(6)]LHRH (6.9 μmol/kg), and the combination of unconjugated DOX and [D-Lys(6)]LHRH (Fig.1A). Tumor growth was already significantly decreased compared to control at week 1 (71% inhibition vs control, p<0.01); this remained significant throughout the 8 weeks of treatment. The cytotoxic LHRH analog completely blocked tumor growth at the end of the treatment resulting in a 90.5% inhibition compared to control and was also significantly better than [D-Lys(6)]LHRH (p<0.01) and DOX alone (p<0.05). The unconjugated DOX, [D-Lys(6)]LHRH or their combination were unsuccessful in inducing significant reduction in tumor growth. As revealed by Western blot analysis of tumors removed at the end of the treatment, AEZS-108 was also highly potent in elevating protein levels of p21, a key activator of tumor suppressor pathways (Fig.1B).

### Immunocytochemical localization of LHRH receptors in DU-145 cells

**Figure 2:**
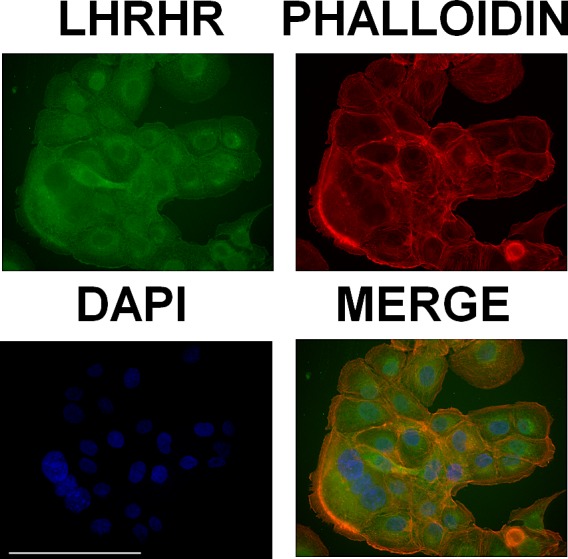
DU-145 cells express receptors of LHRH on the cell membrane LHRH receptors were labelled by using immunocytochemistry (green), the actin cytoskeleton was visualized by TRITC-conjugated phalloidin (red) and nuclei were stained with DAPI (blue). Scale bar corresponds to 100 μm.

Expression and cellular distribution of LHRH receptors in castration-resistant DU-145 cells was revealed by immunocytochemistry (Fig.2). We found that LHRH receptors are present on the cell membrane and therefore they can act to facilitate the selective uptake of AEZS-108 in these cells.

### AEZS-108 inhibits proliferation and increases apoptotic cell death in DU-145 cells

Cell density was measured after a two-day incubation with 100 nM or 250 nM doxorubicin (DOX), [D-Lys(6)]LHRH) (LHRH), the combination of DOX and LHRH or AEZS-108, respectively, by the MTS assay. DOX and its combination with LHRH reduced the proliferation by 14.8% (p<0.01) and 11.3% (p<0.05) at 100 nM concentration, respectively, and 52% and 42.8% at 250 nM concentration (p<0.001), respectively, compared to control. LHRH on its own had little effect on proliferation. The cytotoxic compound, AEZS-108 on the other hand was the most efficacious in inhibiting proliferation, reducing it by 23.2% and 61.2% at 100 nM and 250 nM (p<0.001), respectively, compared to control. The increase in apoptotic cell death was measured after a 1-day incubation with 1 μM DOX, [D-Lys(6)]LHRH), their combination or AEZS-108, respectively, by measuring the binding of FITC-conjugated Annexin-V. The elevation in apoptotic cell death was only significant when AEZS-108 was used (46% elevation, p<0.05).

**Figure 3:**
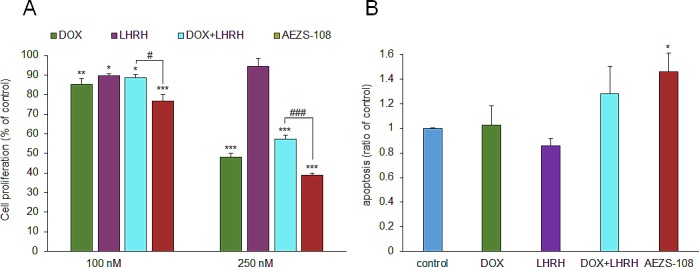
AEZS-108 suppresses the proliferation (A) and induces apoptosis (B) in DU-145 castration-resistant prostate cancer cells *in vitro* A: Cells were split into a 96-well plate at 5000 cells/well density, starved for one day in serum free medium and treated with 100 nM or 250 nM of doxorubicin (DOX), [D-Lys(6)]LHRH (LHRH), the combination of DOX and LHRH or AEZS-108 in 0.5% serum-containing growth medium. B: The density of apoptotic cells were measured by detecting the accumulation of annexin-V after a 1-day treatment with 1 μM doxorubicin (DOX), [D-Lys(6)]LHRH (LHRH), the combination of DOX and LHRH or AEZS-108 diluted in serum-free medium. Statistical analysis was performed by one-way ANOVA, followed by Holm-Sidak test.*: p < 0.05, **: p < 0.01, ***: p < 0.001 vs. control.

### The intracellular distribution of AEZS-108 and the unconjugated doxorubicin are dissimilar

**Figure 4:**
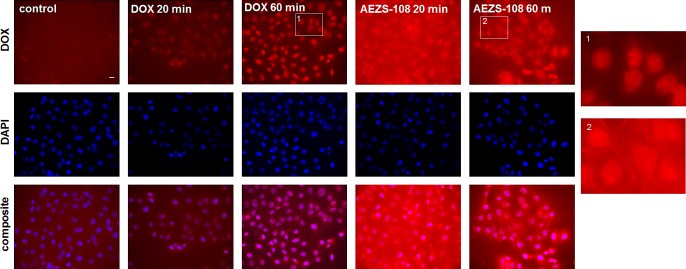
The intracellular distribution of AEZS-108 and doxorubicin are different after a short incubation in DU-145 castration-resistant cells Cells were incubated with 5 μM unconjugated doxorubicin or AEZS-108 for 20 or 60 minutes in serum-free medium. Nuclei were labelled with Hoechst dye (blue). Cells were fixed in 4% paraformaldehyde, mounted and images were acquired immediately by using the inherent fluorescence of doxorubicin. Scale bar corresponds to 10 μm.

The intrinsic fluorescence of doxorubicin was detected in DU-145 cells after they were treated with either 5 μM unconjugated doxorubicin or AEZS-108 (Fig.4.). After 20 or 60 minutes of incubation, the unconjugated doxorubicin was predominantly localized in the nucleus. In contrast, the cytotoxic LHRH compound showed a gradual cellular uptake, accumulating at the cell membrane and in the perinuclear space and showing a limited presence in the nucleus. This indicates that the compound spends extended time outside the nucleus before the subsequently cleaved doxorubicin reaches the nuclear DNA.

### Short-term treatment with AEZS-108 and doxorubicin

**Figure 5:**
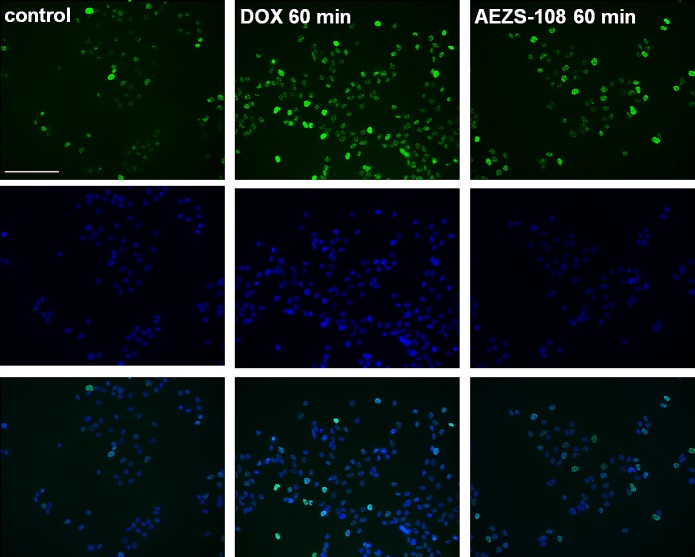
The phosphorylation of the DNA breakdown marker, γ-H2AX, is markedly stimulated by doxorubicin but less affected by AEZS-108 in short-term experiments Cells were incubated with 5 μM unconjugated doxorubicin or AEZS-108 for 60 minutes in serum-free medium. Cells were then fixed in 4% paraformaldehyde and the phosphorylated γ-H2AX was labelled by using immunocytochemistry (green). Nuclei were labelled with DAPI (blue). Scale bar corresponds to 100 μm.

To assess the difference in DNA damage caused by a short term incubation with the unconjugated doxorubicin or AEZS-108, we used immunocytochemistry with an antibody recognizing the phosphorylated form of the histone variant, γ-H2AX (Fig.5). After 60 minutes of incubation, the unconjugated form of doxorubicin was noticeably more efficient than AEZS-108 in increasing the phosphorylation of γ-H2AX. This finding provides evidence for the delayed transport of doxorubicin when it is in conjugated form in the cytotoxic compound, AEZS-108.

### The cytotoxic LHRH analog, AEZS-108, increases the rate of autophagy and the generation of reactive oxygen species (ROS)

**Figure 6:**
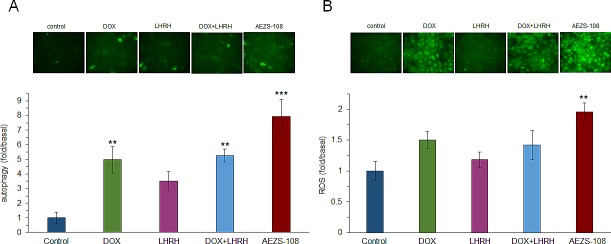
The cytotoxic LHRH analog, AEZS-108, induces autophagy (A) and increases the level of intracellular ROS (B) in the DU-145 castration-resistant prostate cancer cells Cells were incubated with 1 μM doxorubicin (DOX), [D-Lys(6)]LHRH) (LHRH), the combination of DOX and LHRH or AEZS-108 for 24 hours in serum-free medium. The rate of autophagy was measured by labelling autophagosomes with the GFP-tagged p62 reporter transduced by baculovirus. ROS level was detected by APF fluorescent reagent. Fluorescent intensity was measured with a Victor3 plate reader. After measurement, cells were fixed in 4% paraformaldehyde and representative images were taken with a Nikon Eclipse Ti fluorescence microscope with a 20x objective. Statistical analysis was performed by one-way ANOVA, followed by Holm-Sidak test.*: p < 0.05, **: p < 0.01, ***: p < 0.001 vs. control.

The induction of autophagy was measured after 1 day of incubation with 1μM DOX, [D-Lys(6)]LHRH, the combination of DOX and LHRH or AEZS-108, respectively, by using the GFP-tagged p62 reporter construct (Fig.6a). Doxorubicin, [D-Lys(6)]LHRH and their unconjugated combination increased the rate of autophagy by 5, 3.5 and 5.3-fold, respectively, although the effect of [D-Lys(6)]LHRH alone was not significant (p<0.01 for DOX and DOX + [D-Lys(6)]LHRH. More intriguingly, AEZS-108 increased the rate of autophagy by 8-fold (p<0.001). The effect of the same treatments on the generation of ROS was measured by a 3'-(p-aminophenyl) fluorescein. Only the cytotoxic compound AEZS-108 increased the level of ROS significantly (2-fold increase, p<0.01).

### AEZS-108 and doxorubicin regulate the protein level of p21 and the phosphorylation of γ-H2AX differently

Protein levels of p21 and the phosphorylation of γ-H2AX were measured after 1 day of incubation with 1μM DOX, [D-Lys(6)]LHRH, their unconjugated combination or AEZS-108, respectively, in DU-145 cells. Interestingly, p21 level was elevated by AEZS-108 to a much higher extent than was achieved by the incubation with the unconjugated DOX and [D-Lys(6)]LHRH combination. In contrast, the phosphorylation of γ-H2AX was similar in the DOX, DOX+[D-Lys(6)]LHRH and AEZS-108 treated cells, indicating that the doxorubicin moiety subsequently cleaved from AEZS-108 also reached and caused damage in the nucleus. Histone H3 and β-actin were used as loading controls.

**Figure 7:**
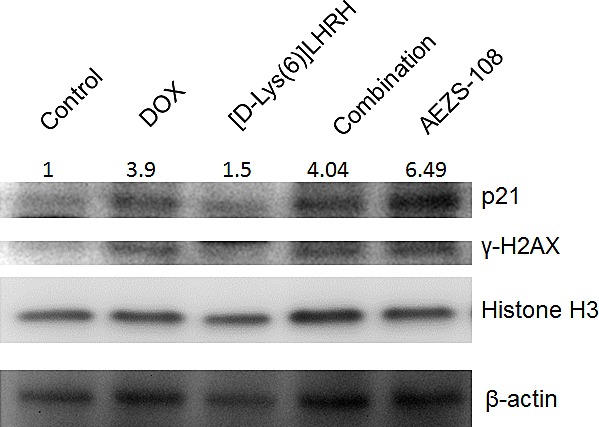
AEZS-108 elevates the protein level of p21 WAF1/Cip1 but its effect on the phosphorylation of γ-H2AX is comparable to that of unconjugated doxorubicin Cells were incubated with 1 μM doxorubicin (DOX), [D-Lys(6)]LHRH) (LHRH), the combination of DOX and LHRH or AEZS-108 for 24 hours in serum-free medium and then were lysed and were subjected to Western blot analysis. A Histone H3 and β-actin were used as loading controls. Fold-changes in p21 protein levels are shown relative to control and normalized to β-actin levels. Membrane shown is representative of two experiments.

## DISCUSSION

Cytotoxic conjugates of peptide analogs assist the tumor-specific targeting of cytotoxic drugs thereby increasing efficacy and limiting toxicity to the non-tumoral tissue [27, 44-50]. LHRH was selected for the development cytotoxic conjugates based on the presence of its receptor in various tumoral tissues including ovarian, endometrial, colorectal, pancreatic, bladder and prostate cancer as well as in glioblastoma [13, 28-30, 51-53]. More than 80% of resected human prostate cancers express LHRH receptors suggesting that patients with prostatic malignancies would benefit from therapy with the cytotoxic LHRH analog, AEZS-108 [23].

In an earlier study, AEZS-108 suppressed the growth of the human androgen sensitive MDA-PCa-2b and LNCaP cells as well as increased the rate of apoptosis in the castration-resistant bone metastasis model of C4-2 prostate cancer cells [29]. Our aim here was to further elucidate the antitumor effects of AEZS-108 in castration-resistant prostate cancer, a tumor that otherwise lacks effective treatment strategies. We selected an established cell line, DU-145, that is derived from a brain metastasis and manifests an epithelial nature [54], to compare the inhibitory activity of AEZS-108 with its unconjugated constituents, doxorubicin and [D-Lys(6)]LHRH. We found that the effect of AEZS-108 was much greater than that of the doxorubicin alone, or its combination with the unconjugated LHRH in completely blocking and reversing tumor growth. This high efficacy of the cytotoxic analog AEZS-108 is likely due to its accumulation in LHRH receptor positive DU-145 cells, since the combination of unconjugated DOX and [D-Lys(6)]LHRH was much less effective in suppressing tumor growth.

In previous studies, AEZS-108 was also more effective than equimolar doses of doxorubicin to inhibit the growth of experimental human breast, bladder, pancreatic, ovarian and endometrial cancers [13, 51, 53, 55, 56]. These experimental reports led to the first clinical trials that were carried out starting in 2008 [57]. A phase-I dose escalation study was completed in women with LHRH receptor positive ovarian, endometrial and breast cancer [32] and was followed by two phase II studies in patients with taxane-pretreated platinum-resistant ovarian cancer and with recurrent endometrial cancer [58, 59]. These studies showed no major toxicity in pituitary, heart or other organs [58, 59]. AEZS-108 was more efficient in patients with recurrent endometrial cancer: 5% of patients had a complete and 18% a partial remission; moreover, in 44%, the disease was stable for at least 6 weeks [59]. A phase I study with taxane- and castration-resistant prostate cancer has also been finished rendering AEZS-108 safe for its use in phase II and III trials that are currently ongoing (unpublished).

The increased antitumoral activity of the cytotoxic LHRH analog resides in its ability to home the doxorubicin moiety to LHRH receptor positive cancer cells where it undergoes binding and internalization [41, 42]. By using immunocytochemistry, we confirmed that the DU-145 castration-resistant prostate cancer cells express LHRH receptors that are localized on the cell membrane rendering them susceptible to AEZS-108. In addition, we showed that there is a slow uptake of AEZS-108 in DU-145 cells, an effect, which may be responsible for its high efficacy *in vitro*. After a short, one-hour incubation with the cytotoxic analog, AEZS-108 was present at both the cell membrane and in the perinuclear area whereas doxorubicin diffused through the membrane to accumulate in the nucleus. Similar uptake dynamics of AEZS-108 have been shown in breast, ovarian and endometrial cancer cells by others, however, the authors have not compared the uptake of AEZS-108 to that of the unconjugated doxorubicin, and the significance of this extended extranuclear residence has not been discussed [41, 60].

Since the accumulation of doxorubicin derived from AEZS-108 in the nucleus is delayed, it is unlikely that the increased activity of the compound is due to its elevated effect on topoisomerase-II activity. This is supported by our finding that the phosphorylation of the DNA-breakdown marker, γH2AX, is lower after a short incubation with AEZS-108 than with the unconjugated doxorubicin. An alternative cytotoxic mechanism for doxorubicin is considered to be its capability to raise the levels of ROS which process occurs mainly in the cytoplasm and mitochondria [61-63]. Accordingly, doxorubicin has been shown to increase the level of hydrogen peroxide in PC3 prostate cancer cells [64]. Since AEZS-108 remains extranuclear for an extended time, it is expected to influence intracellular ROS levels to a greater extent than the unconjugated DOX. Indeed, we found that AEZS-108 elevated ROS levels more significantly than the unconjugated DOX. In addition, higher ROS levels may contribute to the increased apoptotic effect of AEZS-108 seen in our experiment [65].

Bioactive sphingolipids such as sphingosine, sphingosine-1-phosphate and ceramide are important regulators of survival, cell proliferation and death [35]. Increased levels of ceramide affect the activity of diverse signaling molecules leading to cell cycle arrest, autophagy and apoptosis [36]. Recently, doxorubicin has been shown to promote the synthesis of ceramide [37-39]. Accumulation of ceramide induced by doxorubicin stimulates the proteolytic activation of CREB3L1, a transcription factor responsible for the transcription of various genes that inhibits cell proliferation, including p21 [38, 40]. The extended presence of AEZS-108 on cell membranes may facilitate the synthesis of ceramide and it may thereby produce a greater effect on this pathway than the unconjugated doxorubicin which simply diffuses across membranes [33, 34]. This idea seems to be supported by the increased rate of autophagy and the elevated levels of p21 in response to AEZS-108 in our experiments, both processes being regulated partly by the ceramide pathway [36]. However, further investigation is required to better understand the role of this pathway in cytotoxicity induced by AEZS-108.

In conclusion, our results indicate that patients with castration-resistant prostate cancer may benefit from chemotherapy based on the targeted cytotoxic LHRH analog, AEZS-108. The peptide-conjugated form of doxorubicin allows a slower intracellular uptake of this drug that leads to increased effects on extranuclear targets, such as the generation of ROS and the activation of the ceramide pathway. Our findings provide a better understanding of the mechanism of action of cytotoxic peptide analogs and substantiate their use in clinical practice.

## METHODS

### Cytotoxic compounds

LHRH agonist [D-Lys6]LHRH (pyroGlu-His-Trp-Ser-Tyr-D-Lys-Arg-Pro-Gly-NH2) was synthesized in our laboratory by solid-phase methods as described [66]. DOX.HCl salt was purchased from Chemex Export-Import GmbH (Vienna, Austria). Cytotoxic LHRH conjugate AN-152 was first synthesized in our laboratory [27, 28]. AEZS-108 was made by AEterna/Zentaris (Frankfurt am Main, Germany). The compounds were dissolved in 0.01 M aqueous acetic acid and diluted with 5.5% (w/v) aqueous D-mannitol.

### Cell culture

The DU-145 castration-resistant prostate cancer cell line was obtained from the American Type Culture Collection (ATCC; Manassas, VA) and was maintained in culture using Eagle's Minimum Essential Medium (EMEM; ATCC) supplemented with 10% FBS and antibiotics (100 U/mL penicillin, 100 μg/mL streptomycin). Cells were grown at 37 °C in a humidified 95% air/5% CO_2_ atmosphere.

### Animals and *in vivo* xenograft models of DU-145 tumors

Male athymic nude mice (Ncr nu/nu) were obtained from the NCI (Frederick Cancer Research and Development Center, Frederick, MD), and maintained under pathogen-limited conditions. Four donor animals were injected subcutaneously with 10^7^ DU-145 cells. The resulting tumors were removed aseptically and minced into 3 mm^3^ pieces for transplantation into both flanks of animals that was performed using trocar needles. When tumors reached an average volume of 65 mm^3^, mice were randomized into five groups with nearly equal average tumor sizes. Mice received weekly intravenous injections of 6.9 μmol/kg doxorubicin, [D-Lys(6)]LHRH, their combination, AEZS-108 or vehicle. Tumor size was measured with a microcaliper at each week. Tumor volume [67] and tumor inhibition [68, 69] were calculated as previously described. At the end of the experiment, mice were sacrificed by cervical dislocation and tumors were removed and snap frozen. Specimens were stored at -80°C for further investigation.

### Proliferation assay

Cells were seeded in 96-well plates at 2,500 cells/well density in complete growth medium and were incubated for 24 hrs. After 24 hrs, culture medium was replaced by serum-free medium for another 24 hrs. This was followed by the addition of 100 nM or 250 nM of unconjugated doxorubicin, [D-Lys(6)]LHRH), their combination, or AEZS-108 in medium containing 0.5% heat-inactivated serum; the cells were then incubated for a further 48 h. At the end of the treatment the relative number of viable cells was measured using MTS assay (CellTiter 96 AQueous Assay; Promega, Madison, WI) following manufacturer's instructions. Absorbance was measured at 490 nm in a Victor 3 Multilabel Counter (Perkin-Elmer, Waltham, MD). Experiments were performed in hexaplicate. Values were expressed relative to the control.

### Apoptosis assay

Cells were seeded into a 24-well plate at 100,000 cells/well density in complete growth medium and were incubated for 24 hrs. Afterwards, medium was replaced with serum-free medium containing 1 μM unconjugated doxorubicin, [D-Lys(6)]LHRH), their combination, or AEZS-108. Twenty-four hours later, cells were trypsinized, collected into microcentrifuge tubes and the rate of apoptosis was measured by the Multi-Parameter Apoptosis Assay Kit (Cayman Chemical Company, Ann Arbor, MI) according to the manufacturer's instructions. Experiments were performed in triplicate. Values were expressed relative to the control.

### Detection of ROS

Cells were seeded at 20,000 cells/well density into a 96-well plate in complete growth medium. One μM unconjugated doxorubicin, [D-Lys(6)]LHRH), their combination, or AEZS-108 was added to the cells in serum-free medium for 24 hrs. The ROS sensor reagent, aminophenyl fluorescein (Life technologies, Grand Island, NY) was added to the cells at 5 μM concentration for 20 minutes. Medium was replaced with serum- and phenol red-free medium and fluorescence was measured in a Victor 3 Multilabel Counter (at excitation and emission wavelengths of 485 nm and 535 nm, respectively). Experiments were performed in hexaplicate. Values were expressed relative to the control.

### Measurement of density of autophagic vacuoles

Cells were seeded at 20,000 cells/well density into a 96-well plate in complete growth medium. The next day, cells were transduced by green fluorescent protein-tagged LC3B protein by using the baculovirus technique (Premo™ Autophagy Sensor, Life technologies) according to the manufacturer's instructions. Medium was replaced the next day with serum-free medium containing 1 μM unconjugated doxorubicin, [D-Lys(6)]LHRH), their combination or AEZS-108. Fluorescence was detected in serum- and phenol red-free medium in a Victor 3 Multilabel Counter (at excitation and emission wavelengths of 485 nm and 535 nm, respectively). Experiments were performed in hexaplicate. Values were expressed relative to the control.

### Immunocytochemistry and fluorescence microscopy

DU-145 cells were seeded onto multiwell chamberslides (Millipore, Billerica, MA) and were left to adhere overnight. For the drug-uptake studies, cells were incubated with 5 μM of the unconjugated doxorubicin or AEZS-108 for 20 or 60 minutes, respectively. The nucleus marker Hoechst 33342 dye (Life Technologies) was added 10 minutes before the end of the incubation. Cells were then washed 3 times with PBS, were fixed in 4% paraformaldehyde for 15 minutes, and mounted in Vectashield mounting medium (Vector Laboratories, Burlingame, CA). Fluorescence was detected by a Nikon Eclipse Ti fluorescence microscope (Nikon Instruments, Melville, NY).

For immunocytochemistry, cells were incubated with 5 μM the unconjugated doxorubicin or AEZS-108, respectively, for 60 minutes. Afterwards, cells were washed with PBS 3 times and fixed in 4% paraformaldehyde for 7 minutes. Permeabilization was performed by a 10-minute incubation in PBS containing 0.2% Triton-X and then cells were blocked with 2% goat serum in PBS for 30 min. A specific antibody against the phosphorylated (at Ser139) histone variant γ-H2AX (1:50 dilution, Cell Signalling Technology, Danvers, MA) or the human LHRH receptor (1:100 dilution, Abcam, Cambridge, MA) was diluted in PBS and added to the cells for one hour. Anti-rabbit or anti-goat secondary antibodies (Alexa Fluor 488; Jackson Immunoresearch, West Grove, PA) were also applied for 1 h. Coverslips were mounted in Vectashield mounting medium containing DAPI for nuclear staining (Vector Laboratories). Images were acquired on a Nikon Eclipse Ti fluorescence microscope.

### Western blot

Tumors and cells were homogenized and sonicated in RIPA buffer and debris was removed by centrifugation at 14,000 g for 15 minutes. Protein concentration was determined by BCA protein assay (Thermo Fisher Scientific, Waltham, MA). Western blot analyses were as extensively described [70]. Briefly, equal amount of proteins were mixed with Laemmli buffer (Bio-Rad Laboratories Inc., Hercules, CA) and were incubated at 98°C for 8 minutes on a heat block. Proteins were separated by 4-15% PAGE and were transferred onto PVDF membrane. Blocking was performed with Clear milk blocking buffer (Thermo Fisher Scientific) for half an hour. Primary antibodies p21, γ-H2AX, Histone H3 (1:1000 dilution, Cell Signalling Technology) and β-actin (1:10,000 dilution, Santa Cruz Biotechnology, Inc., Santa Cruz, CA) were added to the membranes for overnight incubation. Species-specific HRP-conjugated secondary antibodies (Thermo Fisher Scientific) were left on the membranes for 1.5 hrs and the signal was developed with ECL reagent (Thermo Fisher Scientific) and then imaged with a Bio-Rad Chemidoc System. Densitometry was performed using ImageJ software (NIH, Bethesda, MD).

### Statistical analysis

Statistical analyses were performed using analysis of variance (ANOVA) followed by Holm-Sidak *post hoc* test calculated by Sigma Plot software (Systat Software Inc, Chicago, IL). Results are expressed as the means ± SEM. Differences with p<0.05, compared to the control, were considered statistically significant.

## References

[1] Siegel R, Ma J, Zou Z, Jemal A (2014). Cancer statistics, 2014. CA: a cancer journal for clinicians.

[2] Rick FG, Block NL, Schally AV (2013). Agonists of luteinizing hormone-releasing hormone in prostate cancer. Expert opinion on pharmacotherapy.

[3] Tennakoon JB, Shi Y, Han JJ, Tsouko E, White MA, Burns AR, Zhang A, Xia X, Ilkayeva OR, Xin L, Ittmann MM, Rick FG, Schally AV, Frigo DE (2013). Androgens regulate prostate cancer cell growth via an AMPK-PGC-1alpha-mediated metabolic switch. Oncogene.

[4] Fahrenholtz CD, Rick FG, Garcia MI, Zarandi M, Cai RZ, Block NL, Schally AV, Burnstein KL (2014). Preclinical efficacy of growth hormone-releasing hormone antagonists for androgen-dependent and castration-resistant human prostate cancer. Proc Natl Acad Sci U S A.

[5] Schally AV (2008). New approaches to the therapy of various tumors based on peptide analogues. Hormone and metabolic research = Hormon- und Stoffwechselforschung = Hormones et metabolisme.

[6] Thompson IM, Zeidman EJ, Rodriguez FR (1990). Sudden death due to disease flare with luteinizing hormone-releasing hormone agonist therapy for carcinoma of the prostate. The Journal of urology.

[7] Cook T, Sheridan WP (2000). Development of GnRH antagonists for prostate cancer: new approaches to treatment. The oncologist.

[8] Shore ND (2013). Experience with degarelix in the treatment of prostate cancer. Therapeutic advances in urology.

[9] Gonzalez-Barcena D, Vadillo-Buenfil M, Gomez-Orta F, Fuentes Garcia M, Cardenas-Cornejo I, Graef-Sanchez A, Comaru-Schally AM, Schally AV (1994). Responses to the antagonistic analog of LH-RH (SB-75, Cetrorelix) in patients with benign prostatic hyperplasia and prostatic cancer. The Prostate.

[10] Rick FG, Block NL, Schally AV (2013). An update on the use of degarelix in the treatment of advanced hormone-dependent prostate cancer. OncoTargets and therapy.

[11] Heidenreich A, Pfister D, Merseburger A, Bartsch G, German Working Group on Castration-Resistant Prostate C (2013). Castration-resistant prostate cancer: where we stand in 2013 and what urologists should know. European urology.

[12] Garcia JA, Rini BI (2012). Castration-resistant prostate cancer: many treatments, many options, many challenges ahead. Cancer.

[13] Jaszberenyi M, Schally AV, Block NL, Nadji M, Vidaurre I, Szalontay L, Rick FG (2013). Inhibition of U-87 MG glioblastoma by AN-152 (AEZS-108), a targeted cytotoxic analog of luteinizing hormone-releasing hormone. Oncotarget.

[14] Rick FG, Saadat SH, Szalontay L, Block NL, Kazzazi A, Djavan B, Schally AV (2013). Hormonal manipulation of benign prostatic hyperplasia. Current opinion in urology.

[15] Rick FG, Schally AV, Block NL, Abi-Chaker A, Krishan A, Szalontay L (2013). Mechanisms of synergism between antagonists of growth hormone-releasing hormone and antagonists of luteinizing hormone-releasing hormone in shrinking experimental benign prostatic hyperplasia. The Prostate.

[16] Rick FG, Schally AV, Block NL, Halmos G, Perez R, Fernandez JB, Vidaurre I, Szalontay L (2011). LHRH antagonist Cetrorelix reduces prostate size and gene expression of proinflammatory cytokines and growth factors in a rat model of benign prostatic hyperplasia. Prostate.

[17] Rick FG, Szalontay L, Schally AV, Block NL, Nadji M, Szepeshazi K, Vidaurre I, Zarandi M, Kovacs M, Rekasi Z (2012). Combining growth hormone-releasing hormone antagonist with luteinizing hormone-releasing hormone antagonist greatly augments benign prostatic hyperplasia shrinkage. J Urol.

[18] Volker P, Grundker C, Schmidt O, Schulz KD, Emons G (2002). Expression of receptors for luteinizing hormone-releasing hormone in human ovarian and endometrial cancers: frequency, autoregulation, and correlation with direct antiproliferative activity of luteinizing hormone-releasing hormone analogues. American journal of obstetrics and gynecology.

[19] Imai A, Ohno T, Iida K, Fuseya T, Furui T, Tamaya T (1994). Gonadotropin-releasing hormone receptor in gynecologic tumors. Frequent expression in adenocarcinoma histologic types. Cancer.

[20] Friess H, Buchler M, Kiesel L, Kruger M, Beger HG (1991). LH-RH receptors in the human pancreas. Basis for antihormonal treatment in ductal carcinoma of the pancreas. International journal of pancreatology : official journal of the International Association of Pancreatology.

[21] Bahk JY, Kim MO, Park MS, Lee HY, Lee JH, Chung BC, Min SK (2008). Gonadotropin-releasing hormone (GnRH) and GnRH receptor in bladder cancer epithelia and GnRH effect on bladder cancer cell proliferation. Urologia internationalis.

[22] Baumann KH, Kiesel L, Kaufmann M, Bastert G, Runnebaum B (1993). Characterization of binding sites for a GnRH-agonist (buserelin) in human breast cancer biopsies and their distribution in relation to tumor parameters. Breast cancer research and treatment.

[23] Halmos G, Arencibia JM, Schally AV, Davis R, Bostwick DG (2000). High incidence of receptors for luteinizing hormone-releasing hormone (LHRH) and LHRH receptor gene expression in human prostate cancers. The Journal of urology.

[24] Buchholz S, Seitz S, Schally AV, Engel JB, Rick FG, Szalontay L, Hohla F, Krishan A, Papadia A, Gaiser T, Brockhoff G, Ortmann O, Diedrich K, Koster F (2009). Triple-negative breast cancers express receptors for luteinizing hormone-releasing hormone (LHRH) and respond to LHRH antagonist cetrorelix with growth inhibition. Int J Oncol.

[25] Hohla F, Winder T, Greil R, Rick FG, Block NL, Schally AV (2014). Targeted therapy in advanced metastatic colorectal cancer: Current concepts and perspectives. World journal of gastroenterology : WJG.

[26] Liu SV, Schally AV, Hawes D, Xiong S, Fazli L, Gleave M, Cai J, Groshen S, Brands F, Engel J, Pinski J (2010). Expression of receptors for luteinizing hormone-releasing hormone (LH-RH) in prostate cancers following therapy with LH-RH agonists. Clinical cancer research : an official journal of the American Association for Cancer Research.

[27] Schally AV, Nagy A (1999). Cancer chemotherapy based on targeting of cytotoxic peptide conjugates to their receptors on tumors. European journal of endocrinology / European Federation of Endocrine Societies.

[28] Schally AV, Nagy A (2004). Chemotherapy targeted to cancers through tumoral hormone receptors. Trends in endocrinology and metabolism: TEM.

[29] Letsch M, Schally AV, Szepeshazi K, Halmos G, Nagy A (2003). Preclinical evaluation of targeted cytotoxic luteinizing hormone-releasing hormone analogue AN-152 in androgen-sensitive and insensitive prostate cancers. Clinical cancer research : an official journal of the American Association for Cancer Research.

[30] Liu SV, Liu S, Pinski J (2011). Luteinizing hormone-releasing hormone receptor targeted agents for prostate cancer. Expert opinion on investigational drugs.

[31] Engel JB, Schally AV, Dietl J, Rieger L, Honig A (2007). Targeted therapy of breast and gynecological cancers with cytotoxic analogues of peptide hormones. Molecular pharmaceutics.

[32] Emons G, Kaufmann M, Gorchev G, Tsekova V, Grundker C, Gunthert AR, Hanker LC, Velikova M, Sindermann H, Engel J, Schally AV (2010). Dose escalation and pharmacokinetic study of AEZS-108 (AN-152), an LHRH agonist linked to doxorubicin, in women with LHRH receptor-positive tumors. Gynecol Oncol.

[33] Nitiss JL (2009). Targeting DNA topoisomerase II in cancer chemotherapy. Nature reviews Cancer.

[34] Tewey KM, Rowe TC, Yang L, Halligan BD, Liu LF (1984). Adriamycin-induced DNA damage mediated by mammalian DNA topoisomerase II. Science.

[35] Ogretmen B, Hannun YA (2004). Biologically active sphingolipids in cancer pathogenesis and treatment. Nature reviews Cancer.

[36] Morad SA, Cabot MC (2013). Ceramide-orchestrated signalling in cancer cells. Nature reviews Cancer.

[37] Dumitru CA, Carpinteiro A, Trarbach T, Hengge UR, Gulbins E (2007). Doxorubicin enhances TRAIL-induced cell death via ceramide-enriched membrane platforms. Apoptosis : an international journal on programmed cell death.

[38] Denard B, Lee C, Ye J (2012). Doxorubicin blocks proliferation of cancer cells through proteolytic activation of CREB3L1. eLife.

[39] Gewirtz DA (1999). A critical evaluation of the mechanisms of action proposed for the antitumor effects of the anthracycline antibiotics adriamycin and daunorubicin. Biochemical pharmacology.

[40] Denard B, Seemann J, Chen Q, Gay A, Huang H, Chen Y, Ye J (2011). The membrane-bound transcription factor CREB3L1 is activated in response to virus infection to inhibit proliferation of virus-infected cells. Cell host & microbe.

[41] Krebs LJ, Wang X, Pudavar HE, Bergey EJ, Schally AV, Nagy A, Prasad PN, Liebow C (2000). Regulation of targeted chemotherapy with cytotoxic lutenizing hormone-releasing hormone analogue by epidermal growth factor. Cancer research.

[42] Speelmans G, Staffhorst RW, Steenbergen HG, de Kruijff B (1996). Transport of the anti-cancer drug doxorubicin across cytoplasmic membranes and membranes composed of phospholipids derived from Escherichia coli occurs via a similar mechanism. Biochimica et biophysica acta.

[43] Gunthert AR, Grundker C, Bongertz T, Schlott T, Nagy A, Schally AV, Emons G (2004). Internalization of cytotoxic analog AN-152 of luteinizing hormone-releasing hormone induces apoptosis in human endometrial and ovarian cancer cell lines independent of multidrug resistance-1 (MDR-1) system. American journal of obstetrics and gynecology.

[44] Schally AV, Comaru-Schally AM, Plonowski A, Nagy A, Halmos G, Rekasi Z (2000). Peptide analogs in the therapy of prostate cancer. The Prostate.

[45] Nagy A, Schally AV (2005). Targeting of cytotoxic luteinizing hormone-releasing hormone analogs to breast, ovarian, endometrial, and prostate cancers. Biology of reproduction.

[46] Hohla F, Buchholz S, Schally AV, Krishan A, Rick FG, Szalontay L, Papadia A, Halmos G, Koster F, Aigner E, Datz C, Seitz S (2010). Targeted cytotoxic somatostatin analog AN-162 inhibits growth of human colon carcinomas and increases sensitivity of doxorubicin resistant murine leukemia cells. Cancer Lett.

[47] Pozsgai E, Schally AV, Halmos G, Rick F, Bellyei S (2010). The inhibitory effect of a novel cytotoxic somatostatin analogue AN-162 on experimental glioblastoma. Horm Metab Res.

[48] Seitz S, Buchholz S, Schally AV, Jayakumar AR, Weber F, Papadia A, Rick FG, Szalontay L, Treszl A, Koster F, Ortmann O, Hohla F (2013). Targeting triple-negative breast cancer through the somatostatin receptor with the new cytotoxic somatostatin analogue AN-162 [AEZS-124]. Anti-cancer drugs.

[49] Seitz S, Schally AV, Treszl A, Papadia A, Rick F, Szalontay L, Szepeshazi K, Ortmann O, Halmos G, Hohla F, Buchholz S (2009). Preclinical evaluation of properties of a new targeted cytotoxic somatostatin analog, AN-162 (AEZS-124), and its effects on tumor growth inhibition. Anticancer Drugs.

[50] Treszl A, Schally AV, Seitz S, Szalontay L, Rick FG, Szepeshazi K, Halmos G (2009). Inhibition of human non-small cell lung cancers with a targeted cytotoxic somatostatin analog, AN-162. Peptides.

[51] Szepeshazi K, Schally AV, Keller G, Block NL, Benten D, Halmos G, Szalontay L, Vidaurre I, Jaszberenyi M, Rick FG (2012). Receptor-targeted therapy of human experimental urinary bladder cancers with cytotoxic LH-RH analog AN-152 [AEZS- 108]. Oncotarget.

[52] Szepeshazi K, Schally AV, Halmos G (2007). LH-RH receptors in human colorectal cancers: unexpected molecular targets for experimental therapy. International journal of oncology.

[53] Szepeshazi K, Schally AV, Block NL, Halmos G, Nadji M, Szalontay L, Vidaurre I, Abi-Chaker A, Rick FG (2013). Powerful inhibition of experimental human pancreatic cancers by receptor targeted cytotoxic LH-RH analog AEZS-108. Oncotarget.

[54] Stone KR, Mickey DD, Wunderli H, Mickey GH, Paulson DF (1978). Isolation of a human prostate carcinoma cell line (DU 145). International journal of cancer Journal international du cancer.

[55] Bajo AM, Schally AV, Halmos G, Nagy A (2003). Targeted doxorubicin-containing luteinizing hormone-releasing hormone analogue AN-152 inhibits the growth of doxorubicin-resistant MX-1 human breast cancers. Clinical cancer research : an official journal of the American Association for Cancer Research.

[56] Miyazaki M, Nagy A, Schally AV, Lamharzi N, Halmos G, Szepeshazi K, Groot K, Armatis P (1997). Growth inhibition of human ovarian cancers by cytotoxic analogues of luteinizing hormone-releasing hormone. Journal of the National Cancer Institute.

[57] Emons G, Sindermann H, Engel J, Schally AV, Grundker C (2009). Luteinizing hormone-releasing hormone receptor-targeted chemotherapy using AN-152. Neuroendocrinology.

[58] Emons G, Gorchev G, Sehouli J, Wimberger P, Stahle A, Hanker L, Hilpert F, Sindermann H, Grundker C, Harter P (2014). Efficacy and safety of AEZS-108 (INN: Zoptarelin Doxorubicin Acetate) an LHRH agonist linked to doxorubicin in women with platinum refractory or resistant ovarian cancer expressing LHRH receptors: A multicenter Phase II trial of the ago-study group (AGO GYN 5). Gynecologic oncology.

[59] Emons G, Gorchev G, Harter P, Wimberger P, Stahle A, Hanker L, Hilpert F, Beckmann MW, Dall P, Grundker C, Sindermann H, Sehouli J (2014). Efficacy and safety of AEZS-108 (LHRH agonist linked to doxorubicin) in women with advanced or recurrent endometrial cancer expressing LHRH receptors: a multicenter phase 2 trial (AGO-GYN5). International journal of gynecological cancer : official journal of the International Gynecological Cancer Society.

[60] Wang X, Krebs LJ, Al-Nuri M, Pudavar HE, Ghosal S, Liebow C, Nagy AA, Schally AV, Prasad PN (1999). A chemically labeled cytotoxic agent: two-photon fluorophore for optical tracking of cellular pathway in chemotherapy. Proceedings of the National Academy of Sciences of the United States of America.

[61] Thorn CF, Oshiro C, Marsh S, Hernandez-Boussard T, McLeod H, Klein TE, Altman RB (2011). Doxorubicin pathways: pharmacodynamics and adverse effects. Pharmacogenetics and genomics.

[62] Pawlowska J, Tarasiuk J, Wolf CR, Paine MJ, Borowski E (2003). Differential ability of cytostatics from anthraquinone group to generate free radicals in three enzymatic systems: NADH dehydrogenase, NADPH cytochrome P450 reductase, and xanthine oxidase. Oncology research.

[63] Doroshow JH (1986). Role of hydrogen peroxide and hydroxyl radical formation in the killing of Ehrlich tumor cells by anticancer quinones. Proceedings of the National Academy of Sciences of the United States of America.

[64] Wagner BA, Evig CB, Reszka KJ, Buettner GR, Burns CP (2005). Doxorubicin increases intracellular hydrogen peroxide in PC3 prostate cancer cells. Archives of biochemistry and biophysics.

[65] White SJ, Kasman LM, Kelly MM, Lu P, Spruill L, McDermott PJ, Voelkel-Johnson C (2007). Doxorubicin generates a proapoptotic phenotype by phosphorylation of elongation factor 2. Free radical biology & medicine.

[66] Nagy A, Schally AV, Armatis P, Szepeshazi K, Halmos G, Kovacs M, Zarandi M, Groot K, Miyazaki M, Jungwirth A, Horvath J (1996). Cytotoxic analogs of luteinizing hormone-releasing hormone containing doxorubicin or 2-pyrrolinodoxorubicin, a derivative 500-1000 times more potent. Proceedings of the National Academy of Sciences of the United States of America.

[67] Rick FG, Schally AV, Szalontay L, Block NL, Szepeshazi K, Nadji M, Zarandi M, Hohla F, Buchholz S, Seitz S (2012). Antagonists of growth hormone-releasing hormone inhibit growth of androgen-independent prostate cancer through inactivation of ERK and Akt kinases. Proc Natl Acad Sci U S A.

[68] Rick FG, Buchholz S, Schally AV, Szalontay L, Krishan A, Datz C, Stadlmayr A, Aigner E, Perez R, Seitz S, Block NL, Hohla F (2012). Combination of gastrin-releasing peptide antagonist with cytotoxic agents produces synergistic inhibition of growth of human experimental colon cancers. Cell Cycle.

[69] Rick FG, Seitz S, Schally AV, Szalontay L, Krishan A, Datz C, Stadlmayr A, Buchholz S, Block NL, Hohla F (2012). GHRH antagonist when combined with cytotoxic agents induces S-phase arrest and additive growth inhibition of human colon cancer. Cell Cycle.

[70] Rick FG, Abi-Chaker A, Szalontay L, Perez R, Jaszberenyi M, Jayakumar AR, Shamaladevi N, Szepeshazi K, Vidaurre I, Halmos G, Krishan A, Block NL, Schally AV (2013). Shrinkage of experimental benign prostatic hyperplasia and reduction of prostatic cell volume by a gastrin-releasing peptide antagonist. Proceedings of the National Academy of Sciences of the United States of America.

